# Non-rigid motion-corrected free-breathing 3D myocardial Dixon LGE imaging in a clinical setting

**DOI:** 10.1007/s00330-022-08560-6

**Published:** 2022-02-20

**Authors:** Martin Georg Zeilinger, Karl-Philipp Kunze, Camila Munoz, Radhouene Neji, Michaela Schmidt, Pierre Croisille, Rafael Heiss, Wolfgang Wuest, Michael Uder, René Michael Botnar, Christoph Treutlein, Claudia Prieto

**Affiliations:** 1 Institute of Diagnostic Radiology, University Hospital of Erlangen, Erlangen, Germany; 2 MR Research Collaborations, Siemens Healthcare GmbH, Frimley, UK; 3 School of Biomedical Engineering and Imaging Sciences, King’s College London, London, UK; 4 Cardiovascular MR Predevelopment, Siemens Healthcare GmbH, Erlangen, Germany; 5 University of Lyon, UJM-Saint-Etienne, INSA, CNRS UMR 5520, INSERM U1206, CREATIS, Saint-Etienne, France; 6 Institute of Radiology, Martha Maria Hospital, Nuremberg, Germany; 7 Escuela de Ingeniería, Pontificia Universidad Católica de Chile, Santiago, Chile

**Keywords:** Three-dimensional imaging, Heart, Magnetic resonance imaging, Myocardium

## Abstract

**Objectives:**

To investigate the efficacy of an in-line non-rigid motion-compensated reconstruction (NRC) in an image-navigated high-resolution three-dimensional late gadolinium enhancement (LGE) sequence with Dixon water–fat separation, in a clinical setting.

**Methods:**

Forty-seven consecutive patients were enrolled prospectively and examined with 1.5 T MRI. NRC reconstructions were compared to translational motion-compensated reconstructions (TC) of the same datasets in overall and different sub-category image quality scores, diagnostic confidence, contrast ratios, LGE pattern, and semiautomatic LGE quantification.

**Results:**

NRC outperformed TC in all image quality scores (*p* < 0.001 to 0.016; e.g., overall image quality 5/5 points vs. 4/5). Overall image quality was downgraded in only 23% of NRC datasets vs. 53% of TC datasets due to residual respiratory motion. In both reconstructions, LGE was rated as ischemic in 11 patients and non-ischemic in 10 patients, while it was absent in 26 patients. NRC delivered significantly higher LGE-to-myocardium and blood-to-myocardium contrast ratios (median 6.33 vs. 5.96, *p* < 0.001 and 4.88 vs. 4.66, *p* < 0.001, respectively). Automatically detected LGE mass was significantly lower in the NRC reconstruction (*p* < 0.001). Diagnostic confidence was identical in all cases, with high confidence in 89% and probable in 11% datasets for both reconstructions. No case was rated as inconclusive.

**Conclusions:**

The in-line implementation of a non-rigid motion-compensated reconstruction framework improved image quality in image-navigated free-breathing, isotropic high-resolution 3D LGE imaging with undersampled spiral-like Cartesian sampling and Dixon water–fat separation compared to translational motion correction of the same datasets. The sharper depictions of LGE may lead to more accurate measures of LGE mass.

**Key Points:**

*• 3D LGE imaging provides high-resolution detection of myocardial scarring.*

*• Non-rigid motion correction provides better image quality in cardiac MRI.*

*• Non-rigid motion correction may lead to more accurate measures of LGE mass.*

## Introduction

In cardiac MRI (CMR), late gadolinium enhancement (LGE) is the standard method for evaluating necrosis and fibrosis after myocardial infarction, as well as for the assessment of several types of non-ischemic cardiomyopathies [1]. LGE contrast is based on the differential distribution of gadolinium in the extracellular space in healthy and affected myocardial tissue [2]. A two-dimensional (2D) inversion recovery fast spoiled gradient-echo or balanced steady-state free precession (bSSFP) sequence acquired in multiple breath-holds is commonly used [3]. However, a much higher resolution and extended myocardial coverage is delivered by 3D imaging, which facilitates the assessment of scar tissue in thinner myocardial structures such as the atria or the right ventricle [4–6]. This approach enables post-acquisition reformatting in any desired plane, which simplifies sequence planning [7]. To prevent prolonged breath-holds, diaphragmatic navigator-based respiratory motion correction and gating have been developed. However, this strategy has the drawback of long and unpredictable scan times and may require the use of a motion model [8, 9]. To address this problem, 1D self-navigation [10] and 2D image navigators (iNAV) [8, 11, 12] have been proposed to provide direct and model-free respiratory motion tracking of the heart [13]. Translational respiratory motion correction in two spatial dimensions, in concert with model-free motion estimation, would enable a much larger gating window, resulting in up to 100% respiratory scan efficiency. Such an approach would lead to shorter and more predictable scan times compared to conventional respiratory motion tracking techniques [8, 9].

The use of iNAV would allow for the correction for translational respiratory motion on a beat-to-beat basis [13]. To account for non-rigid tissue deformations during the breathing cycle, affine and non-rigid respiratory motion correction techniques have been proposed [14–20]. These approaches rely on the so-called respiratory binning to assign the acquired data into several respiratory motion states (bins), followed by correction against a reference position (e.g., end expiration) using the motion estimated from images reconstructed at each bin [20]. Bin-to-bin non-rigid motion correction is usually performed with a matrix formalism approach that directly incorporates the estimated motion during the reconstruction process [15]. Beat-to-beat iNAV-based translational motion correction and bin-to-bin affine or non-rigid respiratory motion correction have been combined successfully in coronary MR angiography [19–21] and other whole-heart applications [22].

An iNAV-based translational motion correction, near isotropic high-resolution 3D LGE sequence with compressed sensing and Dixon water–fat separation, has been recently proposed and successfully tested in routine clinical practice [23]. The same approach has also been recently combined with non-rigid motion and regularized low-rank patch-based reconstruction, enabling isotropic (1.3 mm)^3^ water–fat LGE images with ~8-min scan times [24]. In contrast to the approach described above [23], where reconstruction is performed in-line in the scanner software, regularized non-rigid motion correction requires long (off-line) reconstruction times of ~210 min, potentially limiting its implementation in a clinical setting.

The purpose of the present study was to evaluate the effect on image quality of in-line beat-to-beat translation respiratory motion correction only versus bin-to-bin non-rigid motion correction. We therefore validated an iNAV-based high-resolution 3D LGE sequence with Dixon water–fat separation in concert with non-regularized non-rigid motion-compensated reconstruction implemented in-line as a prototype scanner software in a routine clinical setting.

## Materials and methods

### Patients

The study protocol was approved by the local Review Board and is compliant with Health Insurance Portability and Accountability Act (HIPAA) criteria. All patients signed informed consent forms prior to study enrolment.

Consecutive patients scheduled for CMR including LGE were screened for study participation, and 47 patients were prospectively included. The study population consisted of 17 women and 30 men (mean age: 51 ± 16 years). Data from a subset of these patients was reported previously [23]. Indications for MRI were pericarditis or myocarditis (*n* = 27), history of myocardial ischemia (*n* = 13) and non-ischemic cardiomyopathies such as non-compaction cardiomyopathy (*n* = 2), sarcoidosis (*n* = 2), HCM (*n* = 1), ARVC (*n* = 1), or systemic lupus erythematosus (*n* = 1). Exclusion criteria were contraindications for MRI, including unsafe implants, pregnancy, known allergies to gadolinium-based contrast agents, and impaired renal function (i.e., estimated glomerular filtration rate below 30 ml/min). Underaged patients and those with continuous, absolute arrhythmia, such as permanent atrial fibrillation, were also excluded from the study.

At study entry, mean body mass index (BMI) was 26.8 ± 5 kg/m^2^. Mean body surface area was 2.0 ± 0.3 m^2^. Mean heart rate during the 3D LGE sequences was 69 ± 12 beats/min. Mean left ventricular (LV) ejection function was 53 ± 11%, mean LV end-diastolic volume was 184 ± 68 ml (91 ± 28 ml/m^2^), and mean LV mass was 150 ± 50 g (73 ± 20 g/m^2^).

### CMR protocol

CMR imaging was performed on a 1.5-T MRI system (MAGNETOM Aera, Siemens Healthcare) with dedicated phased-array receiver coils (18-channel body coil; 32-channel spine coil). The exam was carried out in accordance with standardized protocols [25]. bSSFP cine images in 4-, 3-, and 2-chamber view long-axis and contiguous short-axis slices covering both ventricles were performed. For LGE imaging, an intravenous contrast bolus of 0.2 mmol/kg gadobutrol (Gadovist, Bayer) was administered, according to current recommendations [25]. In addition to the institutional standard 2D LGE sequence using T1w fast gradient-echo phase-sensitive inversion recovery (PSIR) sequences, a high-resolution iNAV-3D LGE prototype sequence with Dixon-based fat–water separation was performed. In 24/47 patients, the 3D sequence was performed after the standard 2D LGE sequence, and in 23/47 patients prior to 2D LGE.

### 3D water–fat iNAV LGE sequence

The 3D dual-echo inversion recovery prepared spoiled gradient-echo prototype sequence was performed in free-breathing, covering the whole heart in transverse orientation. The underlying sequence has been previously described [24] (acquisition parameters are listed in Table 1) and validated against a standard 2D LGE sequence using T1w fast gradient-echo phase-sensitive inversion recovery (PSIR) LGE imaging [23].

**Table 1:** Overview of the scan parameters for 3D LGE

	3D LGE
TR	7.2 ms
TE	TE1/TE2 = 2.38/4.76 ms
Receiver bandwidth	496 Hz/px for both echoes
Flip angle	20°
FOV	312 × 312 mm
Matrix	240 × 240
Resolution	1.3 mm^3^ isotropic resolution
Slices per 3D slab	80–104 depending on heart size

The inversion time was determined by a dedicated Look-Locker prototype sequence triggered on every heart beat [24] and ranged around 245 ± 46 ms. Inversion pulses for the 3D LGE acquisition were performed every RR interval. Acquisition was performed with a 3.3 × undersampled variable-density golden step Cartesian trajectory with spiral profile order sampling (VD-CASPR) [26]. Low-resolution, coronal 2D dual-echo iNAVs were acquired immediately before the 3D acquisition. The iNAV trajectory was centric out-in, while the 3D VD-CASPR trajectory was centric in-out, which means that the centers of *k*-space from iNAV and 3D acquisitions were directly adjacent in time.

### Motion-compensated reconstruction

After data acquisition, the undersampled 3D data of both echoes were independently reconstructed using two different methods: (1) iNAV-based translational motion correction with iterative sensitivity encoding (itSENSE [27]) reconstruction (TC), (2) iNAV-based non-rigid respiratory motion-corrected itSENSE reconstruction (NRC).

For both TC and NRC, the opposed-echo iNAV was used for motion estimation by tracking a template (manually selected during acquisition planning) covering the whole heart along the right–left (RL) direction, and the base and mid-part of the heart along the FH direction [28, 29]. Note that the separation of iNAV and 3D acquisitions in time was very small (a few milliseconds between *k*-space centers) compared to the respiratory cycle.

For TC, these motion estimates were used to correct the 3D data from both echoes to a reference position by modulating the *k*-space data with a linear shift toward an average global breathing position [13], and both echoes were then separately reconstructed using the itSENSE reconstruction.

For NRC, foot–head (FH) motion estimates were used to sort both in- and opposed-phase data into five respiratory bins each. Data in all bins were translationally corrected toward the respective bin center using *k*-space linear phase shift. For the opposed-phase echo, bin images were reconstructed [30] at half of the full imaging resolution, histogram-equalized, and registered to the central bin [31] to estimate non-rigid respiratory motion fields with respect to an average breathing position. The motion fields were then interpolated back to the full image resolution and incorporated into separate non-rigid motion-corrected itSENSE reconstructions [20] for both echoes.

After reconstruction of both echoes, Dixon water–fat separation was performed for both TC and NRC methods to obtain the final 3D water–fat LGE images. The whole reconstruction pipelines for both TC and NRC reconstruction frameworks were completely implemented in-line in the scanner software as a prototype (Works-in-progress package (WIP) 1111, Syngo MR E11C, Siemens Healthcare).

### Image quality analysis

The images were analyzed by two board-certified reviewers (M.Z. and C.T., 9 and 6 years of experience in cardiovascular imaging), who were blinded to all patient and other imaging data. The TC and NRC datasets (in each case water and corresponding in-phase images) were presented randomly in the original axial views and, where necessary, reformatted to other planes using dedicated software (Horos v. 3.3.6, distributed under the LGPL license by Horosproject.org).

Overall image quality and artifacts not related to water–fat separation, such as fat–water swaps (*n* = 2) or insufficient fat suppression, were rated on a 5-point scale for both TC and NRC datasets [32]: 5 = excellent image quality, interpretable with no artifacts; 4 = good image quality, interpretable with minimal artifacts; 3 = average image quality, interpretation mildly degraded by image artifacts; 2 = below average image quality, interpretable but moderately degraded; 1 = poor image quality, uninterpretable images. Chest wall image quality, anatomical details (i.e., depiction of small structures such as trabeculae and valves) and border sharpness between myocardium and blood pool (LV and RV), and LGE and epicardial fat were evaluated on a similar scale (i.e., 5 = excellent, 4 = good, 3 = average, 2 = below average, 1 = poor). If present, the reason for impaired image quality was evaluated.

In order to judge the effectiveness of the two motion correction algorithms in different respiratory patterns, the iNAV images were scored visually in “regular respiration” and “irregular respiration.” These values were correlated to the differences in image quality scores between TC and NRC.

### Contrast ratios

Contrast ratios based on the signal intensities (SI) of LGE respective blood pool and myocardium were compared.

The mean SI of LGE and myocardium (based on three regions of interest [ROIs] each) were quantified in the corresponding representative original axial slices. The mean blood signal intensity was measured luminally in the atria or ventricles without including cardiac structures. Artifacts were avoided when drawing ROIs. All ROIs were copied to the exactly corresponding position from the NRC dataset to the TC dataset.

### LGE pattern and extent

LGE was defined as visually hyperenhanced myocardium compared to remote myocardium. LGE was characterized as subepicardial, patchy, midmyocardial, and diffuse, involving RV insertion points or pericardial. LGE involving the subendocardium in a coronary distribution was rated ischemic.

Diagnostic confidence for the presence of LGE was assessed on a 3-point scale: 2: LGE absent/present with confidence, 1: probable LGE, 0: inconclusive.

Overall LGE mass was quantified using the full width half maximum (FWHM) method using the CMRSegTools plugin (Creatis [33]). FWHM uses half of the maximal signal intensity within the scar as a threshold to determine the scar extent [34].

To avoid bias by segmentation, all endocardial and epicardial contours were copied to the exactly corresponding position from the NRC dataset to the TC dataset. All contours have been drawn on the original axial slices, verified, and corrected in the case of slightly different reference positions due to the respective motion correction methods.

### Statistical analysis

Interval-level data were evaluated for normal distribution using the Shapiro-Wilks test. Data are presented as mean ± standard distribution (SD) or as median and range. Paired *t*-test and Wilcoxon’s signed-rank test were used for comparisons. Spearman’s coefficient for rank calculation (rho) was calculated in order to correlate the differences in image quality to different respiratory patterns. Significance was accepted for *p* values < 0.05. The Bland-Altman plots were calculated to display contrast ratios and LGE mass.

Inter-rater agreement was evaluated by using Cohen’s kappa value (*κ*). *κ* was interpreted as follows [35]: 0 < *κ* ≤ 0.2 = slight agreement, 0.2 < *κ* ≤ 0.4 = fair agreement, 0.4 < *κ* ≤ 0.6 = moderate agreement, 0.6 < *κ* ≤ 0.8 = substantial agreement, 0.8 < *κ* ≤ 1.0 = almost perfect agreement, and *κ*=1 as perfect agreement. All statistical analysis was performed using Scistat (MedCalc Software).

## Results

The mean 3D LGE scan duration was 10:28 ± 1:41 min under free-breathing. The mean delay between contrast injection and the beginning of the 3D LGE acquisition was 19:56 ± 6:39 min. Reconstruction times with graphic processor unit support were around 1 min for TC and 2–3 min for NRC.

### Image quality

Visual grading was significantly better in NRC for overall and chest wall image quality, detection of anatomical details, and border sharpness between myocardium and blood pool (LV and RV), and LGE and epicardial fat (*p* < 0.05 in all cases). Inter-rater agreement was at least substantial in all cases (> 0.6).

Twenty-five out of forty-seven (53%) TC datasets and 11/47 (23%) NRC datasets were downgraded by at least one point in overall image quality because of residual respiratory motion (water-only and in-phase). We did not find any significant correlation between respiratory irregularity and differences in both overall image quality (rho = 0.129, *p* = 0.43) and anatomical details scores (rho = −0.073, *p* = 0.66). In no case did NRC deliver inferior image quality compared to that of TC. In images rated below 5 points, the NRC algorithm showed improvement over TC in 48–88% of image quality subscores. It was especially effective in the “anatomical details” and “border sharpness myocardium-LGE” categories, with improvement in 76% and 88% of datasets rated below 5 points.

**Fig. 1:**
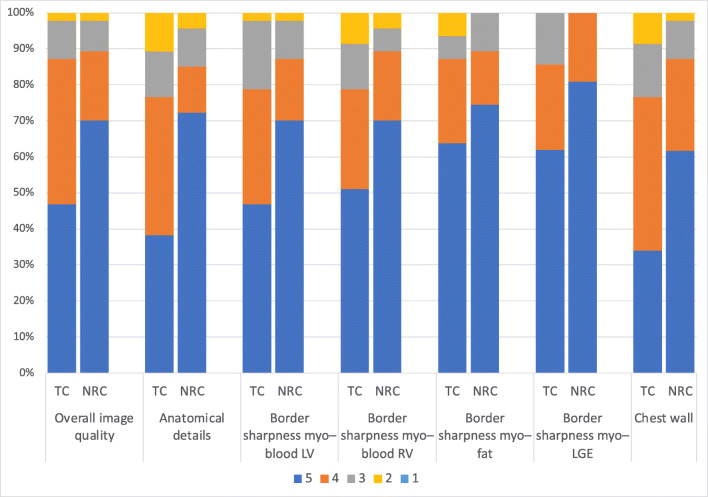
TC (translational motion corrected) and NRC (combined translational and non-rigid motion corrected) 3D Dixon LGE imaging. Detailed scores (reader MZ) are provided for overall image quality, anatomical details (depiction of small structures such as the trabeculae, papillary muscles, and valves), border sharpness myocardium–blood pool (LV, left ventricle, RV, right ventricle), border sharpness myocardium–epicardial fat, border sharpness myocardium–LGE, and chest wall image quality. Scoring as in Table 2

Independently of the reconstruction method, insufficient myocardial nulling occurred in 3/47 (6%) cases. Table 2 and Fig. 1 show image quality scores.

**Table 2:** Summary of subjective and objective image quality scores

	**TC**	**NR**	***p***	**휅 TC**	**휅 NR**
**Overall image quality**	4 (range 2–5)	5 (range 2–5)	< 0.001	0.79	0.78
**Anatomical details**	4 (range 2–5)	5 (range 2–5)	< 0.001	0.65	0.68
**Border sharpness myocardium–blood LV**	4 (range 2–5)	5 (range 2–5)	< 0.001	0.76	0.64
**Border sharpness myocardium–blood RV**	5 (range 2–5)	5 (range 2–5)	< 0.001	0.73	0.67
**Border sharpness myocardium–fat**	5 (range 2–5)	5 (range 3–5)	< 0.001	0.61	0.62
**Border sharpness myocardium–LGE**	5 (range 3–5)	5 (range 4–5)	0.016	0.65	0.83
**Quality chest wall**	4 (range 2–5)	5 (range 2–5)	< 0.001	0.70	0.66
**Contrast ratio blood-to-myocardium**	4.66 (range 1.68–7.44), IQR 2.11	4.88 (range 1.83–8.28), IQR 2.48	0.001		
**Contrast ratio LGE-to-myocardium**	5.96 (range 2.76–13.28), IQR 4.41	6.33 (range 3.22–18.23), IQR 4.94	< 0.001		
**LGE mass at FWHM [g]**	8.19 (range 3.24–30.98), IQR 8.88	6.52 (range 2.15–25.99), IQR 7.77	< 0.001		

*TC*, translational motion corrected; *NRC*, combined translational and non-rigid motion corrected; *FWHM*, full width at half maximum. **Overall image quality scores:** 5 = excellent image quality, interpretable with no artifacts; 4 = good image quality, interpretable with minimal artifacts; 3 = average image quality, interpretation mildly degraded by image artifacts; 2 = below average image quality, interpretable but moderately degraded; 1 = poor image quality, uninterpretable images. **Anatomical details, sharpness, chest wall scores:** 5 = excellent, 4 = good, 3 = average, 2 = below average, 1 = poor. Most data are not normally distributed or ordinal level, so median, range, and interquartile range (IQR) are provided for better comparability

### Contrast ratios

**Fig. 2:**
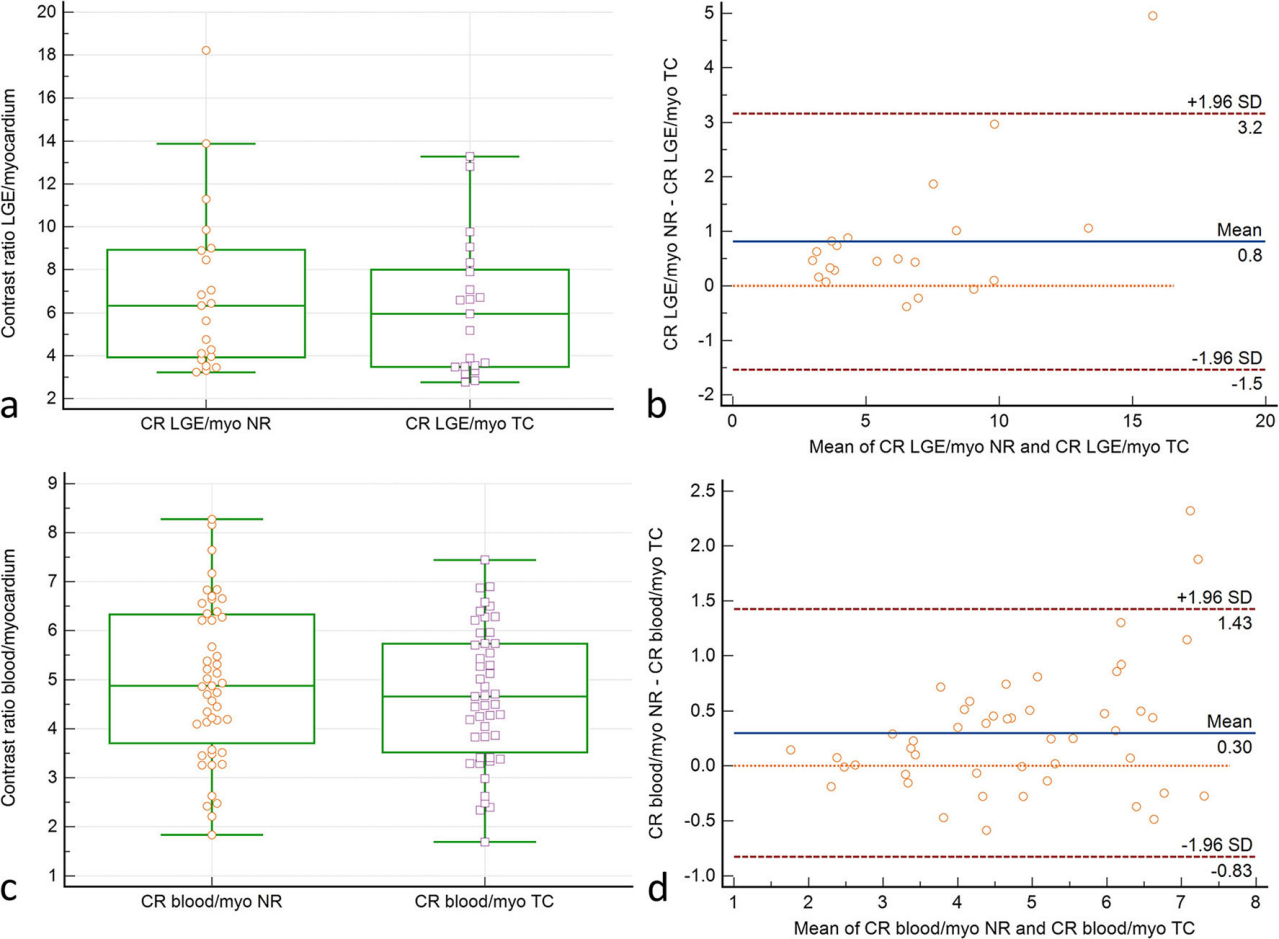
Pairwise comparison of LGE–myocardium (**a**, **b**) and blood–myocardium (**c**, **d**) contrast ratios in the translational (TC) and combined translational/non-rigid (NRC) motion-corrected datasets. Box plots (**a**, **c**): Indicated are the median values (middle line), 25 and 75 percentiles (central box), and single values (dots). The whiskers extend from the minimum to the maximum value, excluding outliers that are outside the lower/upper quartile minus/plus 1.5 times the interquartile range. **a**, **c** Higher LGE–myocardium and blood–myocardium contrast ratios in NRC (*p* < 0.001 resp. 0.001). Bland-Altman plots (**b**, **d**): ∆contrast ratio is shown on the ordinate. The mean of TC and NRC contrast ratios is shown on the abscissa. Indicated are the limits of agreement (± 1.96* SD), the line of equality, and the mean difference. NRC contrast ratios are generally higher than TC contrast ratios. This is particularly true for LGE–myocardium contrast ratios

Both the LGE-to-myocardium and blood-to-myocardium contrast ratios were significantly higher in the NRC datasets (*p* < 0.001, Fig. 2).

### LGE pattern and extent

**Fig. 3:**
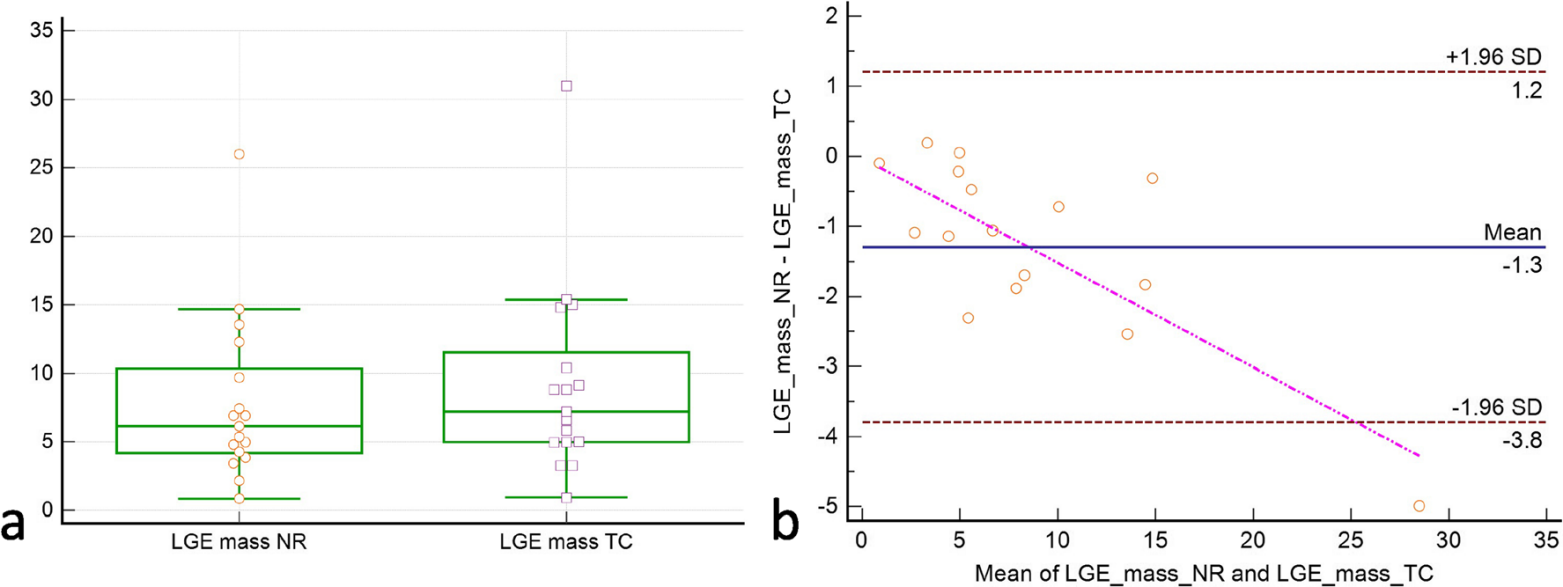
Pairwise comparison of myocardial LGE mass [g] in the translational (TC) and combined translational/non-rigid (NRC) datasets. Box plot (**a**): Indicated are the median values (middle line), 25 and 75 percentiles (central box), and single values (dots). The whiskers extend from the minimum to the maximum value, excluding outliers that are outside the lower/upper quartile minus/plus 1.5 times the interquartile range. Bland-Altman plot (**b**): ∆mass is shown on the ordinate. The mean of NRC and TC LGE masses is shown on the abscissa. Indicated are the limits of agreement (± 1.96* SD) and the mean difference. NRC delivers generally lower LGE masses. The outlier in both datasets was attributed to an exceptionally large infarction (NRC: 25.99 g, TC: 30.98 g) and might indicate a certain proportionality (see the regression line in **b**). The sample size is too small, however, to confirm this observation

In both TC and NRC datasets (water-only and in-phase), the etiological classification of LGE was identical. LGE was rated as ischemic in 11 patients and non-ischemic in 10 patients. No LGE was observed in 26 patients. LGE mass was significantly lower in the NRC reconstruction (TC: median 8.19 (range 3.24–30.98), interquartile range (IQR) 8.88, NRC: median 6.52 (range 2.15–25.99), IQR 7.77, *p* < 0.01, Fig. 3). Diagnostic confidence was identical in all cases, with high confidence (score = 2) in 42/47 (89%) and probable (score = 1) in 5/47 (11%) datasets for TC and NRC (water-only and in-phase) reconstructions. No case was rated as inconclusive (score = 0) with both sequences.

**Fig. 4:**
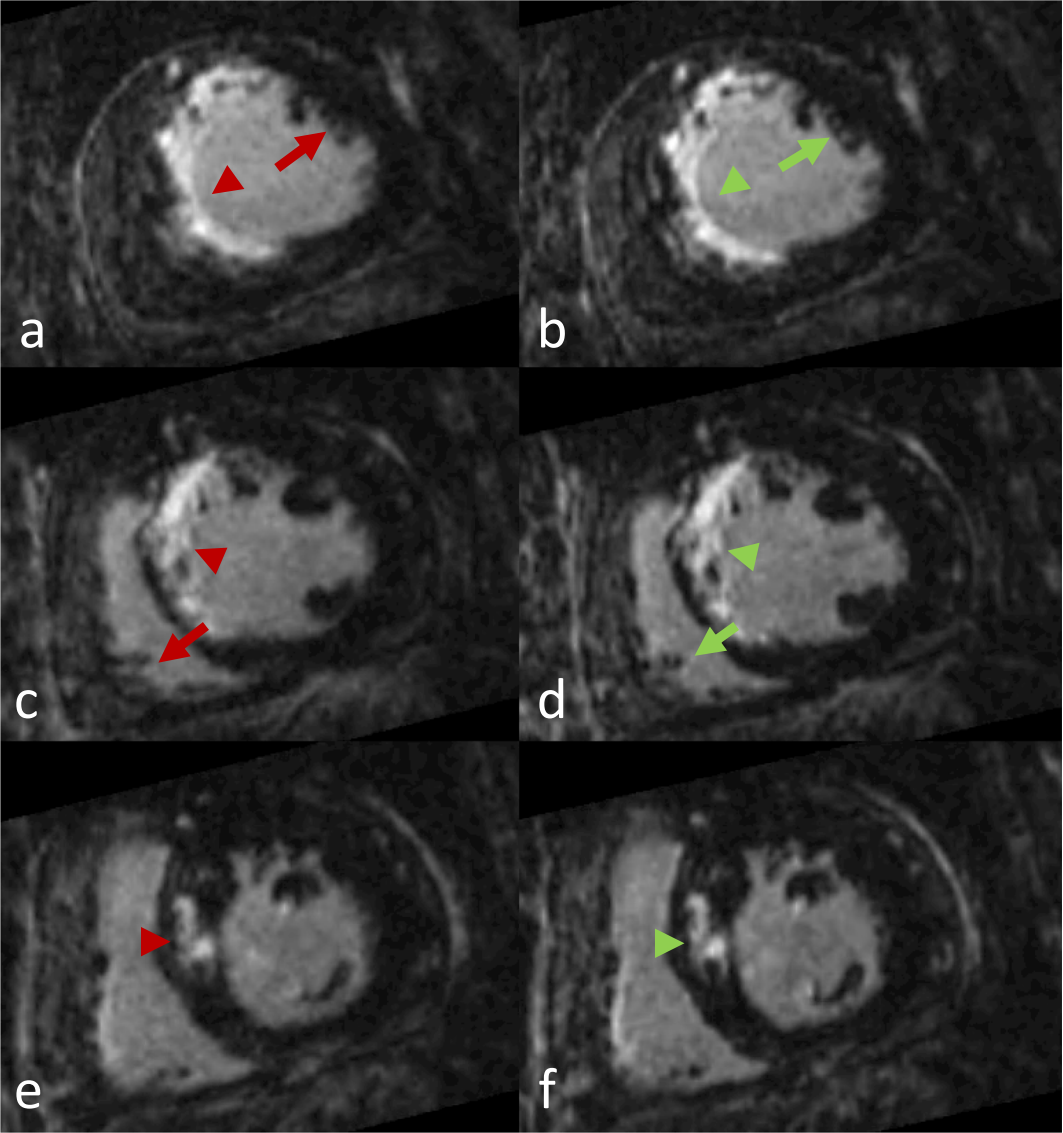
Short-axis reformations in a patient with large septal infarction. Left column: TC (translational motion corrected). Right column: NRC (combined translational and non-rigid motion corrected). Axial (**a**, **b**) and corresponding midventricular (**c**, **d**) and basal (**e**, **f**) short-axis reformations. Excellent overall image quality was observed in both datasets. Note, however, the better LGE delimitation (arrowheads) and anatomical details (arrows) in the NRC reconstruction

**Fig. 5:**
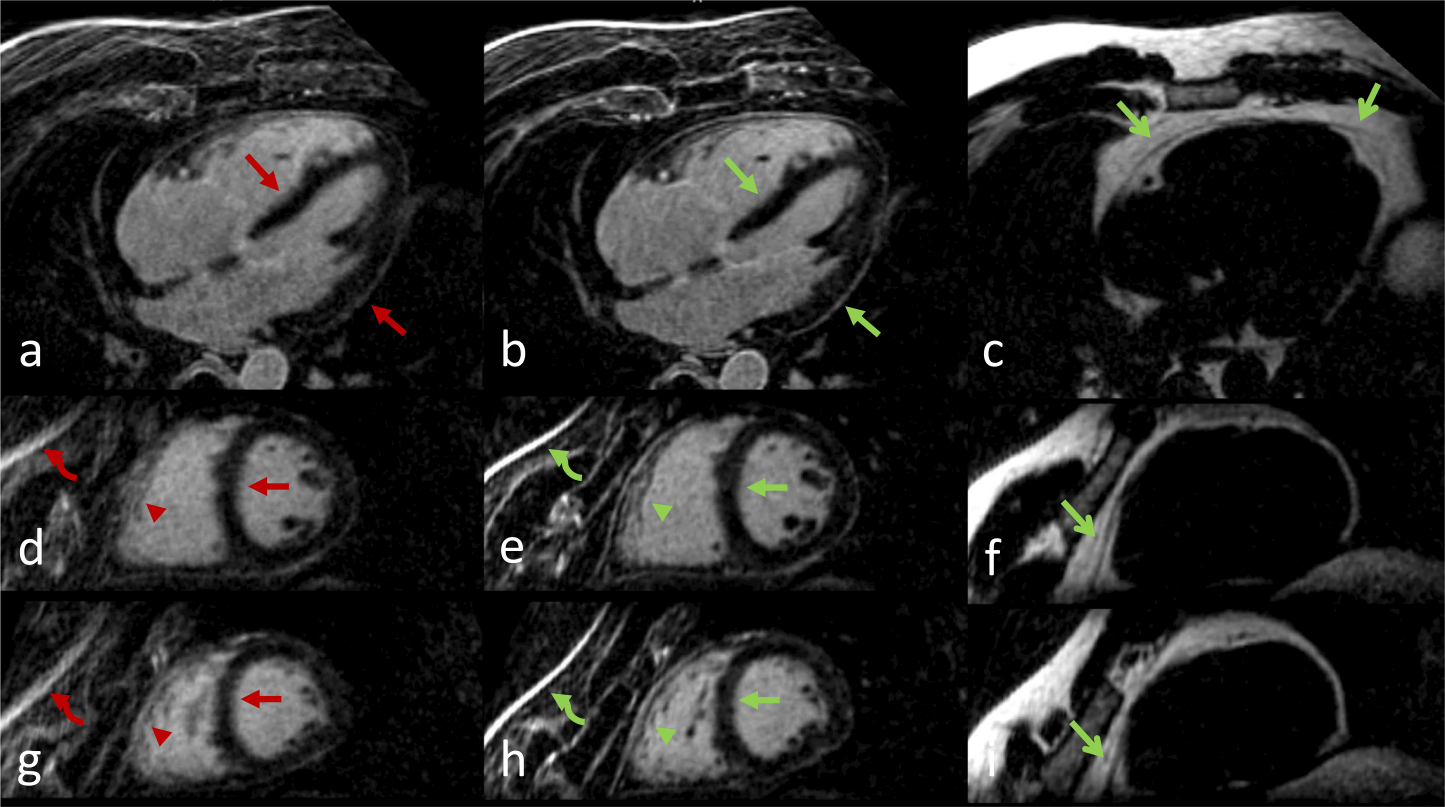
A patient without LGE. **a**–**c** Axial views, **d**–**i** short-axis views. Left column: TC (translational motion corrected). Middle column: NRC (combined translational and non-rigid motion corrected). Right column: Corresponding fat images of NRC. Better overall image quality of the NRC reconstruction was observed. Improved border sharpness of the left ventricular wall (closed arrows), superior anatomical details such as right ventricular trabeculae (arrowheads) and better chest wall image quality (curved arrow) are noticeable in the NRC reconstructions. Note also the fine depiction of the pericardium in the fat images (open arrows)

**Fig. 6:**
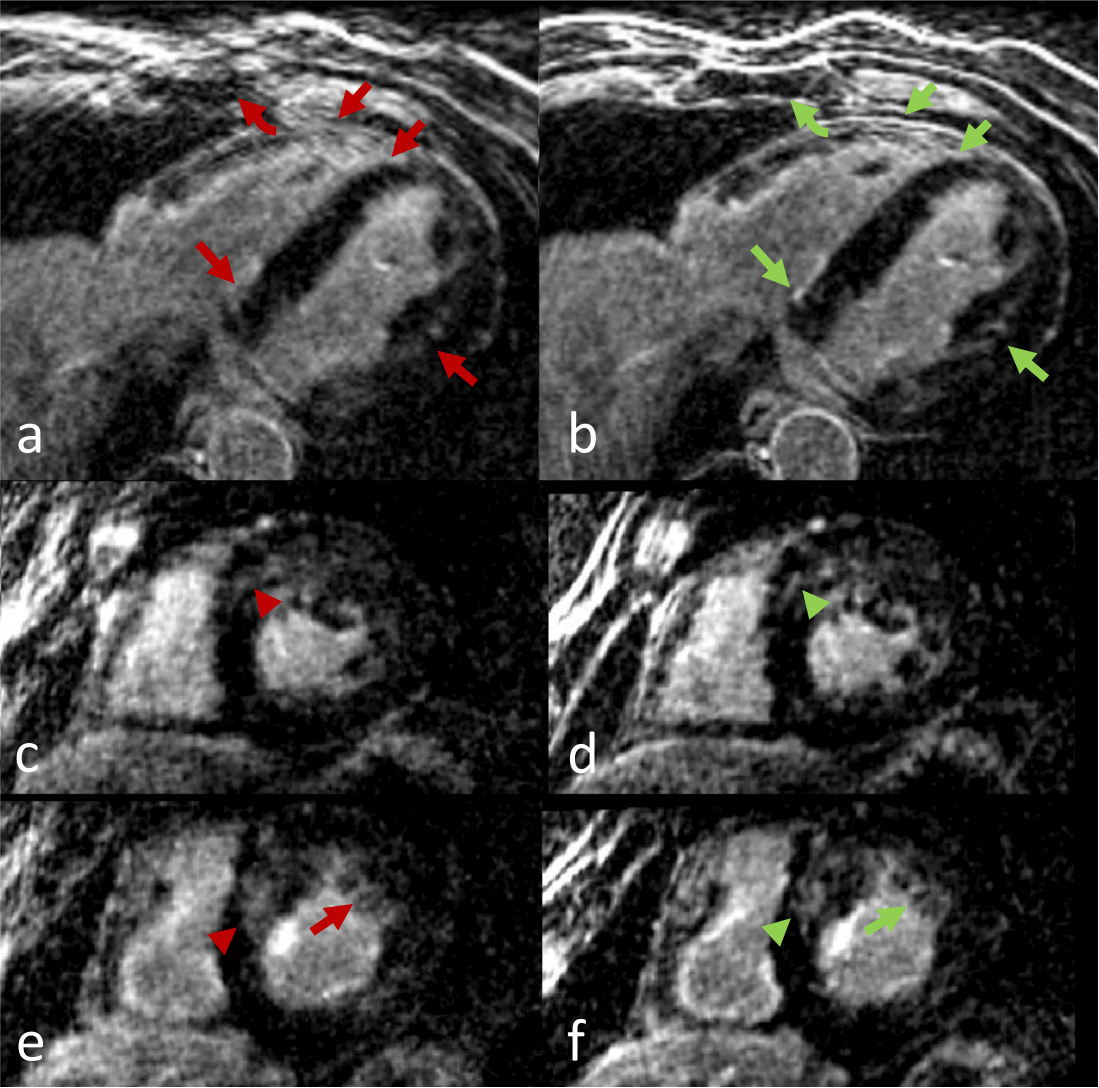
Axial and short-axis views of a patient with hypertrophic cardiomyopathy. Left column: TC (translational motion corrected), right column: NRC (combined translational and non-rigid motion corrected). Improved overall image quality in the NRC reconstruction was observed. Improved depiction of the septum and left ventricular wall with better delineation of papillary muscles and trabeculae (arrows), sharper delineation of basal septal LGE (arrowheads), and better chest wall image quality (curved arrow) can be appreciated in the NRC reconstructions

See Table 3 for detailed LGE patterns and Figures 4, 5, and 6 for examples.

**Table 3:** Distribution of LGE pattern in the *n* = 47 cohort. An identical LGE pattern was observed in both TC (translational motion corrected) and NRC (combined translational and non-rigid motion corrected) reconstructions

LGE pattern	No LGE	Ischemic	Patchy	Subepicardial	Mid wall	Pericardial
*n* = 47	26	11	2	0	3	5

## Discussion

In this study, a novel in-line prototype implementation of an iNAV-based non-rigid (NRC) respiratory motion-corrected reconstruction in high-resolution 3D LGE imaging with Dixon water–fat separation was tested in a routine clinical setting. The proposed approach was compared to a previously introduced iNAV-based translational correction (TC) reconstruction of the same datasets (previously validated against conventional 2D LGE imaging [23]). Overall and chest wall image quality, detection of anatomical details and border sharpness between the myocardium and blood pool (LV and RV), LGE, and epicardial fat were depicted significantly better in the NRC reconstruction (both Dixon fat suppression and in-phase images). Overall image quality was downgraded in only 23% of the NRC datasets vs. 53% of the TC datasets due to residual respiratory motion while 0% were rated as inconclusive. Automatically detected LGE mass was significantly lower in the NRC reconstruction.

Non-rigid motion-compensated reconstructions can be applied to various cardiac applications, with promising 2D examples in LGE imaging [36], 2D cardiac perfusion [37], T2 dark blood [38], and T1 mapping [39]. A more recent 3D application is T2 mapping [40]. Different implementations of the same non-rigid motion-compensated reconstruction framework as the one evaluated in this study have been described before for 3D coronary MR angiography, where it outperformed translational correction alone in terms of image quality [20], as well as showed comparable image quality to that of a reference gated acquisition in a significantly shorter and predictable scan time [28]. In order to potentially achieve higher acceleration factors, regularized versions of the referenced motion-compensated reconstruction framework have been described for CMRA [41] or LGE [24] applications, but typically come at the cost of clinically impractical reconstruction times for in-line implementation.

The application of the non-rigid motion-compensated reconstruction framework concept to 3D LGE imaging delivers superior image quality and border sharpness as demonstrated in [24] and in the present study. This approach may offer significant advantages in the evaluation of LGE for thinner structures such as the atria, the right ventricle, or the epicardium; for example, in the planning and follow-up of atrial and right ventricular ablations [4, 42, 43]. Non-rigid motion correction can be applied not only to the heart but also to the complete field of view, as demonstrated by better NRC chest wall image quality, which, if uncorrected, could lead to ghosting all over the image (Fig. 6). Therefore, it is expected that, both in terms of sharpness as well as for artifact reduction, non-rigid motion correction is especially beneficial in cases with significant motion in the anterior-posterior direction (e.g., “chest-breathing”). This is, however, not easily verifiable using the data at hand as the anterior-posterior direction is not captured by the iNAV, as shown by the weak correlation between iNAV image irregularity and the differences in image quality scores of TC and NRC.

The lower LGE mass and the higher LGE–myocardium contrast ratio in the NRC reconstruction are attributed to reduced blurring and therefore sharper delineation of LGE. This finding is highlighted by a significantly better border sharpness between LGE and myocardium. As such, it may be assumed that the NRC reconstruction reduces the overestimation of scar mass. This finding is in line with previous reports of lower scar mass in (0.91 mm)^3^ high-resolution 3D LGE reconstructions as compared to 1.46 mm × 1.46 mm × 10 mm normal resolution reconstructions of the same datasets [44]. These refinements could enable a better depiction of specific patterns of small LGE lesions, especially critical in non-ischemic cardiomyopathy or as guidance for ablation therapy in arrhythmia patients [44].

The potential change of inversion time due to contrast washout during prolonged scan times in 3D LGE acquisitions has been described as challenging [42]. In our observation, insufficient myocardial nulling was a minor concern and occurred in only 6%.

Other aspects of the evaluated sequence have been discussed in previous reports [23, 24], including 100% respiratory scan efficacy, which allows for a predictable scan time; (1.3 mm)^3^ isotropic resolution that permits individual reformatting; coverage of both ventricles and atria; and robust Dixon water–fat separation which leads to better discrimination of enhanced pericardium and epicardial fat, among others.

### Limitations

Some limitations of the study should be mentioned. As the focus of the study was to detect the effects of combined non-rigid motion correction compared to translational motion correction alone, there is no comparison of contrast ratios and LGE mass with 2D LGE as reference standard. The gold standard for myocardial scar mass quantification is autopsy, which was not ethically justifiable in this study. The sample size for patients showing LGE was relatively small, and patients with permanent, absolute arrhythmia were not included. Studies including this patient cohort should be performed in the future to evaluate the performance of the combined motion correction and arrhythmia rejection in these patients. Image quality assessment was based on subjective scales. Further refinements such as deep learning-based estimation of inter-bin motion as already implemented in coronary MR angiography [45] may further improve image quality, acquisition, and reconstruction time, and will be investigated in future work.

### Conclusion

The described in-line implementation of a non-rigid motion-compensated reconstruction framework improves image quality in iNAV-based free-breathing, isotropic high-resolution 3D LGE imaging with undersampled spiral-like Cartesian sampling and Dixon water–fat separation compared to a 2D iNAV translational motion correction of the same datasets in a realistic clinical setting. The sharper delimitation of LGE allows for better visualization of LGE and may lead to more accurate measures of LGE mass due to sharper border depiction.

## Data Availability

The data supporting the findings of this study are available on reasonable request from the corresponding author. Please note, however, that we are not allowed to make the complete image datasets publicly available as they contain personal information that could compromise the study participants’ privacy and consent.

## Abbreviations

ARVD: Arrhythmogenic right ventricular dysplasia
bSSFP: Balanced steady-state free precession
CMR: Cardiac magnetic resonance imaging
iNAV: Image navigation
LGE: Late gadolinium enhancement
NRC: Non-rigid motion-compensated reconstruction
PSIR: Phase-sensitive inversion recovery
TC: Translational motion-compensated reconstruction
TE: Echo time
TR: Repetition time

## Declarations

### Guarantor

The scientific guarantor of this publication is MZ.

### Statistics and biometry

No complex statistical methods were necessary for this paper.

### Study subjects or cohorts overlap

Some study subjects or cohorts have been previously reported in [23, 24].

### Methodology

• prospective

• diagnostic study

• performed at one institution
